# Bridging exercise biology and immunometabolism: A novel irisin pathway toward next-generation metabolic and neurodegenerative therapies

**DOI:** 10.1016/j.metop.2026.100466

**Published:** 2026-04-09

**Authors:** Maria Dalamaga

**Affiliations:** Department of Biological Chemistry, School of Medicine, National and Kapodistrian University of Athens, 75 Mikras Asias, 11527, Athens, Greece

**Keywords:** Irisin, IL-33, Regulatory T cells, Adipose tissue, Immunometabolism, Obesity, Insulin resistance, Exercise mimetics, Neurodegenerative disorders

## Abstract

Recent work by Mu et al. identifying irisin as a modulator of adipose tissue IL-33 and regulatory T cells introduces a new paradigm in immunometabolic biology, shifting attention from thermogenesis alone toward immune–stromal crosstalk as a determinant of metabolic health. By inducing IL-33 production in adipose mesenchymal stromal cells, irisin preserves ST2+ regulatory T cells (Tregs) in visceral adipose tissue, thereby restraining inflammation, improving insulin sensitivity, and promoting metabolic homeostasis. This mechanism expands the concept of exercise-induced metabolic protection by highlighting adipose tissue immune niches as critical targets of myokine action. In parallel, emerging evidence from preclinical models indicates that irisin-driven IL-33 signaling in subcutaneous adipose tissue contributes to thermogenic activation through mechanisms distinct from Treg-mediated immune regulation, highlighting depot-specific effects of this pathway.

Beyond adipose tissue, irisin has emerged as a pleiotropic mediator with reported roles in glucose homeostasis, cardiovascular protection, and neurobiology. Importantly, accumulating evidence indicates that irisin may also exert neuroprotective effects, including the induction of brain-derived neurotrophic factor (BDNF), amyloid-β (Aβ) clearance, and α-synuclein degradation, thereby linking metabolic and neurodegenerative pathways.

Although the findings of Mu et al. derive from preclinical models, they provide a conceptual model for therapeutic strategies aimed at reproducing selected benefits of exercise in obesity, metabolic and neurodegenerative disorders. Notably, these effects appear to depend on sustained irisin exposure in preclinical systems, supporting a role for irisin as a regulator of long-term immunometabolic homeostasis. Collectively, these observations position the irisin/IL-33/Treg axis as a promising link between exercise, adipose tissue immunity, and systemic metabolic regulation, suggesting that targeting immunometabolic circuits, rather than energy balance alone, may open new avenues for future therapeutic intervention.

## 1 Introduction

We have long known that exercise is medicine. What we have not known is precisely how a molecule derived from contracting muscle can reprogram the immune landscape of adipose tissue to restore metabolic health. In January 2012, Boström and colleagues at Harvard Medical School identified a novel myokine secreted by skeletal muscle during exercise, which they named irisin after Iris, the Greek messenger goddess who served as a link between the gods and humanity [1]. This nomenclature proved prescient:irisin has emerged as a molecular messenger connecting physical activity to metabolic health, immune regulation, and brain function. The study published by Mu et al. in *Nature Metabolism* demonstrating that irisin ameliorates obesity and insulin resistance through adipose tissue IL-33 and regulatory T cells adds a critical immunometabolic dimension to the therapeutic potential of this molecule [2]. This discovery arrives at a time when the growing burden of metabolic and neurodegenerative diseases demands innovative therapeutic strategies beyond conventional pharmacotherapy.

## 2 Discovery and the muscle-fat axis

The discovery of irisin arose from investigations into how exercise mediates systemic metabolic benefits. Boström et al. demonstrated that PGC-1α expression in muscle stimulates FNDC5, a transmembrane protein whose ectodomain is cleaved and secreted as irisin [1]. This 112-amino acid peptide acts on white adipose cells to stimulate UCP1 expression and a broad program of brown-fat-like development through p38 MAPK and ERK signaling, increasing energy expenditure without changes in movement or food intake [1,3]. The finding that even modest elevations in circulating irisin levels improve obesity and glucose homeostasis suggested early therapeutic potential for metabolic disease.

## 3 From thermogenesis to the immunometabolic circuit: A paradigm shift

The current *Nature Metabolism* study fundamentally redefines our understanding of exercise as an immunometabolic intervention. By establishing that irisin regulates adipose tissue immune homeostasis through the IL-33/ST2+ regulatory T cell (Treg) axis, this work positions myokines not only as metabolic modulators but as pleiotropic signaling molecules coordinating inter-organ crosstalk between energy metabolism, inflammation, and tissue homeostasis [2,4,5].

Visceral adipose tissue (VAT), epididymal white adipose tissue (eWAT, the murine visceral depot), and inguinal white adipose tissue (iWAT, the murine subcutaneous depot) exhibit distinct immunometabolic properties. In VAT, Treg cells are functionally specialized populations expressing distinct transcriptional programs, including IRF4, BATF, and PPARγ that distinguish them from lymphoid Tregs [6,7]. These tissue-resident immune cells are not passive bystanders but active metabolic regulators that restrain stromal adipocyte precursor differentiation through oncostatin-M secretion, maintaining tissue architecture and insulin sensitivity [8]. IL-33, acting through the ST2 receptor, is essential for VAT-Treg development and maintenance, with exogenous IL-33 administration reversing obesity-induced Treg deficits and improving insulin resistance in experimental models [6,9].

**Fig. 1:**
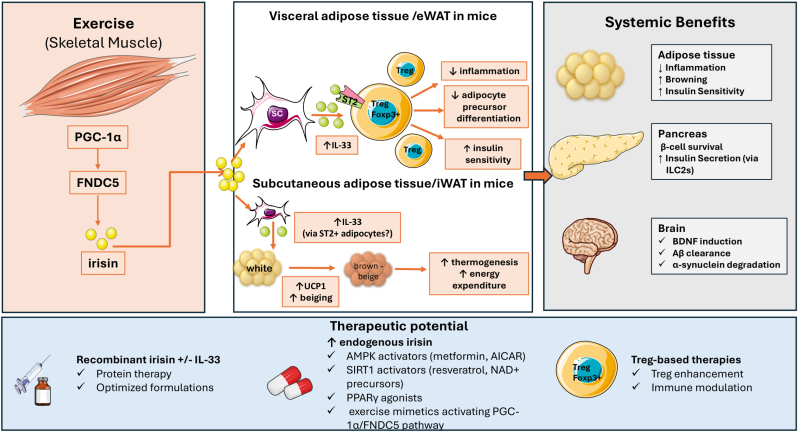
The proposed irisin/IL-33/Treg axis in metabolic regulation and its potential therapeutic implications. Exercise induces PGC-1α-dependent expression of FNDC5 in skeletal muscle, which is cleaved to release irisin into the circulation. In adipose tissues, irisin acts on mesenchymal stromal cells to induce IL-33 production through integrin-mediated signaling pathways. In visceral adipose tissue (corresponding to eWAT in mice), IL-33 promotes the expansion and maintenance of ST2+ Foxp3+ regulatory T cells (Tregs), leading to suppression of inflammation and improved insulin sensitivity. In contrast, in subcutaneous adipose tissue (corresponding to iWAT in mice), IL-33 contributes to the activation of thermogenic programs, including increased UCP1 expression, beiging/browning, and energy expenditure. These effects appear to involve direct or indirect actions on ST2-expressing adipocytes or other resident cells and are mechanistically distinct from the Treg-mediated immunoregulatory pathway observed in visceral fat. These depot-specific effects highlight the context-dependent roles of the irisin/IL-33 axis in coordinating immune regulation and energy homeostasis. Beyond adipose tissue, irisin exerts systemic effects including pancreatic β-cell survival and insulin secretion, as well as neuroprotective actions such as BDNF induction, amyloid-β clearance, and α-synuclein degradation. The lower panel illustrates potential therapeutic strategies targeting this pathway, including recombinant irisin and/or IL-33 administration, pharmacological enhancement of endogenous irisin production, exercise mimetics, and Treg-based immunomodulatory approaches. This schematic is based primarily on preclinical murine data, and the extent to which these mechanisms translate to human physiology remains to be established. The figure was created by Dalamaga M using Servier Medical Art (smart.servier.com), licensed under a Creative Commons Attribution 3.0 Unported License. Abbreviations: Aβ: amyloid-β; BDNF: brain-derived neurotrophic factor; eWAT: epididymal white adipose tissue; FNDC5: fibronectin type III domain-containing protein 5; IL-33: interleukin-33; iWAT: inguinal white adipose tissue; PGC-1α: peroxisome proliferator-activated receptor gamma coactivator 1-alpha; SC: stromal cell; ST2: suppression of tumorigenicity 2; Treg: regulatory T cell.

In this context, the findings by Mu et al. provide a mechanistic link between exercise-derived irisin and IL-33–dependent immune regulation in adipose tissue. Specifically, irisin induces IL-33 production by mesenchymal stromal cells in VAT, leading to preservation of ST2+ Treg cells and attenuation of obesity-associated inflammation. Notably, these immunological effects are spatially restricted, as irisin predominantly modulates immune homeostasis in visceral fat while promoting thermogenic gene programs in subcutaneous depots [2] (Fig. 1, proposed mechanism based on preclinical data). Notably, recent evidence indicates that irisin-induced thermogenesis in subcutaneous adipose tissue (iWAT) is, at least in part, dependent on IL-33 signaling. In contrast to VAT, where IL-33 primarily regulates ST2+ Treg cells and inflammation, IL-33 in subcutaneous depots appears to act through distinct mechanisms, potentially involving direct effects on ST2-expressing adipocytes or stromal cells, thereby promoting thermogenic gene expression [2]. This depot-specific divergence underscores the context-dependent roles of the irisin/IL-33 axis across adipose tissue compartments.

Beyond adipose tissue, the therapeutic implications of this pathway extend to multiple organs. IL-33 rescues perivascular adipose tissue anticontractile function in obesity, thereby normalizing blood pressure through eosinophil-dependent mechanisms [10]. In pancreatic islets, IL-33 promotes β-cell survival under diabetogenic conditions via PPARγ-dependent pathways involving enhanced glucose uptake, mitochondrial function, and anti-inflammatory responses [11]. IL-33 also activates islet-resident innate lymphoid cells that promote insulin secretion through myeloid cell retinoic acid production [12]. Collectively, these observations suggest that the irisin–IL-33 axis may operate as a systems-level regulator of metabolic homeostasis.

## 4 IL-33 as a therapeutic target: overcoming the expression-function paradox

IL-33 therapy has shown remarkable preclinical efficacy, but clinical translation must address the so-called "expression-function paradox" observed in obesity. Despite increased IL-33 expression in obese adipose tissue, protective effects are impaired by effector cell dysfunction, soluble ST2-mediated neutralization, and alterations in downstream signaling pathways [13]. Recent preclinical studies have shown that IL-33 treatment reduces visceral fat mass, improves glucose and insulin tolerance, decreases hepatic steatosis, and modulates inflammatory responses across multiple tissues [14]. Mechanistically, IL-33 promotes adipocyte lipolysis via activation of the β-adrenergic receptor/cAMP/PKA/HSL signaling, providing an additional mechanism beyond immune modulation [15]. In light of these findings, the work by Mu et al. suggests that irisin may act upstream to restore effective IL-33 signaling in adipose tissue, thereby overcoming this functional impairment [2]. The challenge lies in converting abundant but functionally impaired IL-33 into therapeutically effective signaling by essentially reactivating a circuit that obesity has rendered dysfunctional.

## 5 Regulatory T cell modulation: precision immunotherapy

Treg-based approaches represent a frontier in metabolic immunotherapy but require careful consideration of context-dependent effects. Oral anti-CD3 antibody combined with β-glucosylceramide induces TGF-β-dependent Tregs that decrease pancreatic islet hyperplasia, hepatic fat accumulation, and adipose inflammation while improving glucose homeostasis in obese mice [16]. In parallel, recent evidence indicates that hypothalamic Tregs modulate immune activation in high-calorie environments and may reverse metabolic impairments, suggesting that Treg-based interventions may target both peripheral and central mechanisms of metabolic dysfunction [17].

However, the temporal paradox of Treg biology complicates therapeutic development. While Tregs prevent obesity-associated inflammation in young animals, their age-related accumulation may paradoxically contribute to insulin resistance, with Treg depletion preventing age-related metabolic decline [18,19]. This U-shaped relationship underscores the need for precision medicine approaches, with patient stratification based on age, metabolic status, and baseline Treg population [7].

Within this context, the findings by Mu et al. highlight ST2+ adipose tissue Tregs as a functionally distinct subset that mediates, at least in part, the metabolic benefits of irisin, thereby providing a more targeted entry point for future immunomodulatory strategies [2].

## 6 Irisin therapeutics: navigating the human translation challenge

Recombinant irisin has demonstrated strong preclinical efficacy, particularly under conditions of sustained exposure in experimental models, including approaches using adeno-associated virus-mediated expression, where it induces dose-dependent weight loss in diet-induced obesity models through increased energy expenditure and thermogenesis [2,20]. Beyond adipose tissue browning via the p62/Nrf2/HO-1 pathway, irisin activates AMPK signaling, a master regulator of cellular energy homeostasis, thereby reinforcing its role as a candidate exercise mimetic [21]. Notably, many of these effects have been observed under conditions of chronic irisin elevation in preclinical models, suggesting that irisin may function as a regulator of long-term immunometabolic homeostasis rather than solely an acute exercise-induced signal. Moreover, the cardioprotective effects of irisin, mediated through SIRT1/PGC-1α upregulation, include enhanced fatty acid oxidation, mitochondrial biogenesis, reduced oxidative stress, and prevention of maladaptive cardiac hypertrophy [22].

However, clinical translation faces many challenges. A critical barrier is the mutation in the human FNDC5 start codon from ATG to ATA, which reduces full-length protein production to approximately 1% compared to canonical constructs [23]. This finding initially raised questions about the physiological relevance of irisin in humans, although subsequent studies have demonstrated detectable circulating irisin and consistent exercise-induced elevations [24]. The concept of "irisin resistance" in obesity, defined by elevated circulating levels coupled with impaired receptor signaling, parallels insulin and leptin resistance, and suggests that therapeutic strategies must address receptor sensitivity rather than simply increasing circulating concentrations [[25], [26], [27], [28], [29]].

Current therapeutic approaches include: (1) recombinant irisin administration with optimized formulations; (2) pharmacological enhancement of endogenous irisin production through AMPK activators (metformin, 5-Aminoimidazole-4-carboxamide ribonucleoside), SIRT1 activators (resveratrol, NAD + precursors), and PPARγ agonists that upregulate FNDC5 expression; and (3) exercise mimetics that activate the PGC-1α/FNDC5 pathway, including inorganic nitrate supplementation that mimics exercise-stimulated fiber-type switching and myokine release [[30], [31], [32]]. Importantly, in light of the findings by Mu et al., future therapeutic strategies may need to consider not only increasing irisin levels but also preserving the integrity of downstream IL-33/ST2 signaling pathways within adipose tissue.

## 7 Beyond metabolism: irisin and neuroprotection

The irisin/IL-33/Treg axis may have implications extending beyond metabolic disease. Wrann et al. demonstrated that endurance exercise elevates FNDC5 in the hippocampus and that peripheral delivery of FNDC5 induces expression of brain-derived neurotrophic factor (BDNF) and other neuroprotective genes in the brain, establishing the PGC-1α/FNDC5/BDNF pathway as a molecular mechanism linking exercise to cognitive benefits [33]. Lourenco et al. showed that FNDC5/irisin levels are reduced in Alzheimer's disease hippocampi and cerebrospinal fluid, and that boosting brain levels restores synaptic plasticity and memory in experimental models [34]. Kim et al. recently demonstrated that irisin reduces amyloid-β pathology by increasing astrocytic release of the Aβ-degrading enzyme neprilysin, with integrin αV/β5 acting as the irisin receptor required for this effect [35]. In Parkinson's disease models, irisin prevents α-synuclein–induced neurodegeneration through enhanced endolysosomal degradation pathways [36,37].

Although these neuroprotective effects are mechanistically distinct from those described in adipose tissue, they further support the concept of irisin as a pleiotropic regulator of tissue homeostasis. In this context, it remains to be determined whether IL-33–dependent immune pathways may also contribute to the neurobiological actions of irisin. Emerging evidence further suggests that IL-33 may represent a critical intermediary linking irisin to neuroimmune regulation in the central nervous system. IL-33 is abundantly expressed in brain-resident cells, including astrocytes, oligodendrocytes, and endothelial cells, and has been shown to modulate cognitive function in a context-dependent manner [38]. Notably, moderate IL-33 administration has been shown to improve memory and reverse cognitive deficits in Alzheimer's disease mouse models by promoting microglial Aβ clearance, reducing amyloid burden, and polarizing microglia toward anti-inflammatory phenotypes [39]. However, higher or dysregulated IL-33 levels may promote neuroinflammation and impair cognition. Specifically, intra-hippocampal IL-33 injection at certain doses induced microglial activation, IL-1β overexpression, and spatial memory deficits in mice [40]. These observations raise the possibility that irisin-mediated neuroprotection may, at least in part, involve finely tuned IL-33–dependent pathways in the brain, although this hypothesis remains to be directly tested.

## 8 Combination strategies: synergizing pathways

The combination of exercise with GLP-1 receptor agonist therapy demonstrates synergistic effects on metabolic syndrome severity, abdominal obesity, and inflammation beyond individual treatments, reducing metabolic syndrome z-score and decreasing high-sensitivity C-reactive protein by 43% [41]. This provides proof-of-concept that combining exercise-mimetic pathways with incretin-based therapy may optimize cardiometabolic outcomes.

In this context, irisin represents a mechanistically distinct therapeutic modality. Unlike GLP-1 receptor agonists, which primarily act by reducing appetite and caloric intake [42,43], irisin appears to improve metabolic homeostasis, particularly in the context of sustained exposure, through increased energy expenditure, thermogenesis, and immunometabolic regulation, without significantly altering food intake. This distinction suggests that irisin-based interventions may complement, rather than compete with, incretin-based therapies.

Such mechanistic complementarity raises the possibility of combination strategies targeting both sides of the energy balance equation, reducing caloric intake while simultaneously enhancing energy expenditure and resolving adipose tissue inflammation. In addition, irisin-driven activation of the IL-33/Treg axis may provide anti-inflammatory benefits not directly addressed by current pharmacotherapies, further supporting its role in multi-targeted intervention strategies.

Additional combination strategies are under investigation, including irisin plus IL-33 to simultaneously target thermogenesis and immune modulation, as well as approaches combining myokine-based therapies with Treg enhancement to attenuate adipose tissue inflammation. Multi-targeted anti-inflammatory strategies include combined inhibition of TNF-α, IL-1β, and IL-6, alongside modulation of NF-κB, JAK/STAT, and JNK signaling pathways. Furthermore, natural bioactive compounds, including polyphenols (curcumin, resveratrol, catechins) and omega-3 fatty acids, suppress inflammatory pathways while activating AMPK, thereby offering complementary strategies to pharmacological interventions [44,45].

The broader landscape of exercise-induced myokines offers additional therapeutic targets. Meteorin-like protein (Metrnl), another exercise-responsive myokine, mediates muscle-adipose crosstalk by promoting adipose tissue browning, enhancing insulin sensitivity, and exerting anti-inflammatory effects [46]. FGF21, IL-6 (in its anti-inflammatory context), and adiponectin have been exploited therapeutically as exercise mimetics, with several compounds in clinical development [4]. AdipoR activators represent a particularly promising exercise mimetic class, stimulating AMPK/SIRT1/PGC-1α signaling, which is the same pathway activated by exercise, with crystal structures now available to guide drug design [47]. Pharmacological AMPK activation induces transcriptional responses highly congruent with exercise across skeletal muscle, heart, liver, and adipose tissues, further supporting AMPK as a central therapeutic node [48].

## 9 The path forward: from bench to bedside

The identification of the IL-33/Treg axis as a mediator of irisin's effects provides specific biomarkers for therapeutic monitoring. First, standardization of irisin measurement methods is required to resolve discrepancies in reported circulating levels and to establish reliable dose–response relationships [26]. Candidate biomarkers may include circulating irisin levels measured by validated assays, IL-33 and soluble ST2 concentrations, adipose tissue Treg populations quantified by flow cytometry, and downstream metabolic markers including UCP1 expression, betatrophin levels, and AMPK/SIRT1/PGC-1α pathway activation [4,5]. Moreover, baseline exerkine signatures may help predict individual responses to exercise-mimetic interventions, thereby enabling more personalized therapeutic approaches [4,30].

Both irisin resistance and IL-33 dysfunction in obesity require targeted interventions. Strategies to enhance receptor sensitivity, reduce soluble decoy receptors (sST2), and restore effector cell function will be critical for therapeutic success. A deeper understanding of the molecular basis of these resistance mechanisms, including post-receptor signaling defects and epigenetic modifications, represents an important research priority.

While preclinical data are encouraging, potential adverse effects require careful evaluation. Excessive irisin levels may exert deleterious effects, and the relationship between irisin and bone metabolism is complex, with evidence suggesting potential promotion of bone resorption through integrin receptor antagonism [49]. Long-term safety studies will be essential before widespread clinical implementation, including careful assessment of potential immune modulation associated with sustained Treg activation. Clinical trial design must incorporate appropriate biomarkers, patient stratification strategies, and endpoints that capture the multi-system benefits observed in preclinical models [50].

Finally, the myokine field is rapidly expanding beyond irisin to include Metrnl, FGF21, and other exercise-responsive factors with therapeutic potential. Understanding the spatiotemporal dynamics of myokine secretion, mapping receptor–ligand interaction networks across organs, and developing computational models predicting system-level responses will facilitate the translation of exercise physiology into precision therapeutics.

## 10 Conclusion

From its discovery as a messenger between muscle and fat, irisin has emerged as a pleiotropic hormone with therapeutic potential spanning metabolic disease and neurodegeneration. The demonstration that irisin improves obesity and insulin resistance through the IL-33/Treg axis adds an immunometabolic dimension to its mechanisms of action, highlighting that the benefits of exercise extend beyond direct metabolic effects to include regulation of tissue-resident immune networks.

By linking muscle-derived signals to adipose tissue immune homeostasis, the irisin/IL-33/Treg axis provides a unifying model connecting exercise, inflammation, and metabolic regulation. The convergence of mechanistic insights with therapeutic innovation raises the possibility of selectively reproducing key benefits of physical activity in individuals unable to engage in sufficient exercise, including patients with physical limitations, severe obesity, or advanced metabolic disease. Importantly, these effects may depend on sustained activation of irisin signaling pathways, as suggested by preclinical models of chronic exposure.

Future studies will need to address the challenges of translating these findings into human biology, including issues of irisin resistance, IL-33 signaling dysfunction, and long-term safety. As the goddess Iris carried messages between realms, her molecular namesake may ultimately deliver the benefits of physical activity to patients, potentially transforming the management of obesity, type 2 diabetes, and related cardiometabolic and neurodegenerative disorders.

## Funding

This work did not receive any specific grant from funding agencies in the public, commercial or not-for-profit sectors.

## Declaration of competing interest

No conflict of interest to declare.
